# Supplementary material for: Nucleus- and plastid-targeted annexin 5 promotes reproductive development in Arabidopsis and is essential for pollen and embryo formation
Source: BMC Plant Biol. 2018 Sep 6;18:183. doi: 10.1186/s12870-018-1405-3 (PMC6127919; doi:10.1186/s12870-018-1405-3)
Supplement: Supplementary file 1 — Table S1. List of binary plasmids used in this study. Table S2. Oligonucleotides used for RT-qPCR. Figure S1. Analysis of ANN5 transcript abundance in flowers at anthesis collected from ANN5 RNAi-silenced and overexpressing lines. Figure S2. Phenotypic characteristics of Arabidopsis with altered ANN5 expression cultivated under a 12 h light regime. Table S3. Timing of reproductive development of Arabidopsis genotypes with altered ANN5 expression cultivated under a 12 h light regime. Figure S3. Impact of RNAi-mediated suppression of ANN5 on pollen viability. Figure S4. Ultrastructure of bicellular microgametophytes isolated from Arabidopsis lines with altered ANN5 expression. Figure S5. Growth of pollen tubes in pistils 24 h after hand-pollination. Table S4. List of plastidial proteins co-purified with ANN5-YFP and identified by mass spectrometry. Figure S6. Subcellular localization of RABE1b-GFP in N. benthamiana leaf epidermal cells. Figure S7. Overexpression of ANN5 influences chlorophyll content and alters expression of genes related to chlorophyll metabolism in Arabidopsis seedlings. (PDF 1160 kb) [file 12870_2018_1405_MOESM1_ESM.pdf]

Table S1. List of binary plasmids used in this study.

No	Binary plasmid	Experiment type
1	pPZP-RCS2 *35S:ANN5-YFP*	Transformation of Arabidopsis, overexpression of *ANN5*
2	pAgrikola *35S:ANN5*(GST)-RNAi<br>See ref. [3]	Transformation of Arabidopsis, *ANN5* knockdown
3	pCAMBIA 1302 35S*:GFP*<br>See ref. [4]	Transformation of Arabidopsis, generation of control transgenic Arabidopsis
4	GWB 551 *35S:ANN5-GFP*	Transient expression in *N. benthamiana* to study the subcellular localization of protein
5	GWB 552 *35S:GFP-ANN5*	Transient expression in *N. benthamiana* to study the subcellular localization of protein
6	GWB 441 *35S:RABE1b-YFP*	Transient expression in *N. benthamiana* to study the subcellular localization of protein
7	GWB 441 *35S:ANN5-YFP*	Transient expression in *N. benthamiana* to measure FLIM-FRET
8	GWB 444 *35S:RABE1b-CFP*	Transient expression in *N. benthamiana* to measure FLIM-FRET

Table S2. Oligonucleotides used for RT-qPCR.

Oligo ID	Atg gene code	sequence
*ANN5*_fw	AT1G68090	CTCCTCGAGTCGATGCTGACC
*ANN5*_rev	AT1G68090	GCTCGTTGTGTTGCATTGCGA
*HEMA1*_fw	AT1G58290	[5]
*HEMA1*_rev	AT1G58290	[5]
*GUN4*_fw	AT3G59400	[5]
*GUN4*_rev	AT3G59400	[5]
*GUN5*_fw	AT5G13630	[5]
*GUN5*_rev	AT5G13630	[5]
*CHLI1*_fw	AT4G18480	[6]
*CHLI1*_rev	AT4G18480	[6]
*PsbA*_fw	ATCG00020	[7]
*PsbA*_rev	ATCG00020	[7]
*LHCB1*_fw	AT1G29910	[5]
*LHCB1*_rev	AT1G29910	[5]
*NYE1*_fw	AT4G22920	[8]
*NYE1*_rev	AT4G22920	[8]
*NYC1*_fw	AT4G13250	[8]
*NYC1*_rev	AT4G13250	[8]
*SAG29*_fw	AT5G13170	[8]
*SAG29*_rev	AT5G13170	[8]
*PP2A*_fw	AT1G13320	TATCGGATGACGATTCTTCGTGCAG
*PP2A*_rev	AT1G13320	GCTTGGTCGACTATCGGAATGAGAG
*YLS8*_fw	AT5G08290	TTACTGTTTCGGTTGTTCTCCATTT
*YLS8*_rev	AT5G08290	CACTGAATCATGTTCGAAGCAAGT
*UBC21*_fw	AT5G25760	CTGCGACTCAGGGAATCTTCTAA
*UBC21*_rev	AT5G25760	TTGTGCCATTGAATTGAACCC

Figure S1.

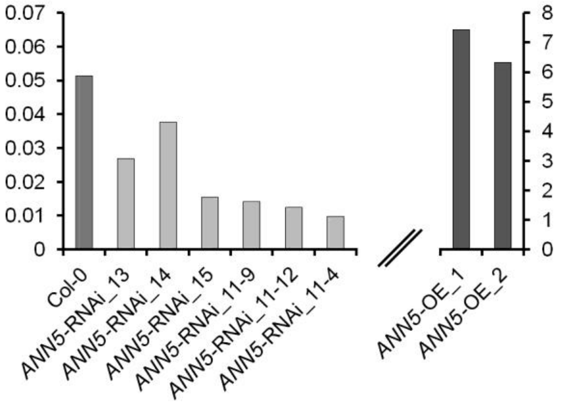

Analysis of *ANN5* transcript abundance in flowers at anthesis collected from *ANN5* RNAi-silenced and overexpressing lines.

Figure S2.

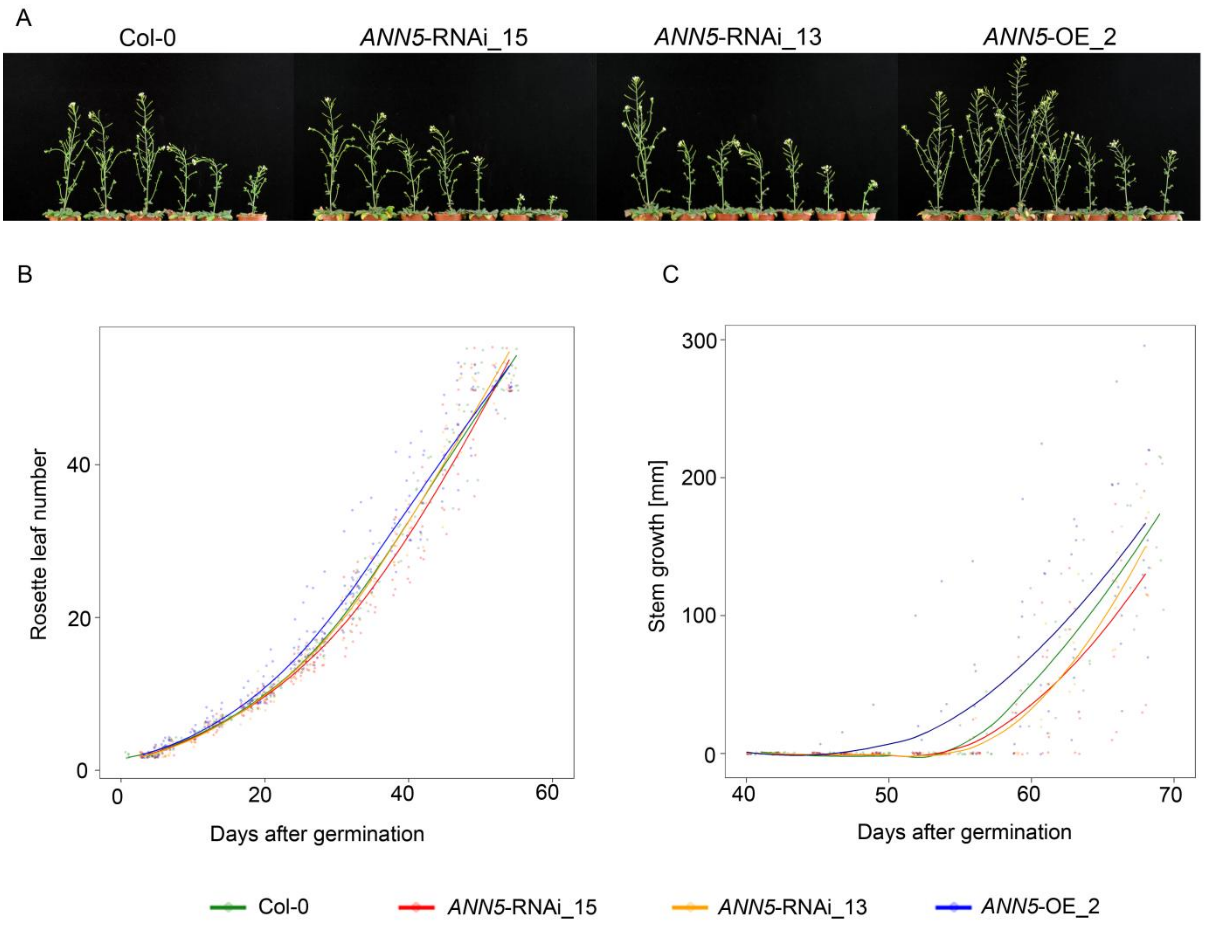

Phenotypic characteristics of Arabidopsis with altered *ANN5* expression cultivated under a 12 h light regime.

(A) Growth of Col-0, *ANN5*-RNAi_15, *ANN5*-RNAi_13, and *ANN5*-OE_2 plants under a 12 h light regime.

(B and C) Comparison of numbers of rosette leaves (B) and length of stems (C) in Col-0, *ANN5*-RNAi_15, *ANN5*-RNAi_13, and *ANN5*-OE_2 plants. Lines represent local regression curves for each genotype. Each developmental stage was recorded for n = 7 individual plants per genotype.

Table S3. Timing of reproductive development of Arabidopsis genotypes with altered *ANN5* expression cultivated under a 12 h light regime.

Genotypes	Bolting	Flowering	Silique formation
	[days]		
Col-0	50.00 ± 1.67	59.80 ± 4.66	65.50 ± 2.66
ANN5-RNAi_13	52.29 ± 2.21	63.43 ± 1.81	67.43 ± 2.76
ANN5-RNAi_15	52.86 ± 3.97	63.83 ± 3.97	67.00 ± 3.41
ANN5-OE_2	50.11 ± 3.79	60.67 ± 4.61	63.67 ± 4.92

Values represent days after germination ± standard error (SE). n = 10 individual plants per line. The data were evaluated with Dunnett' test.

Figure S3.

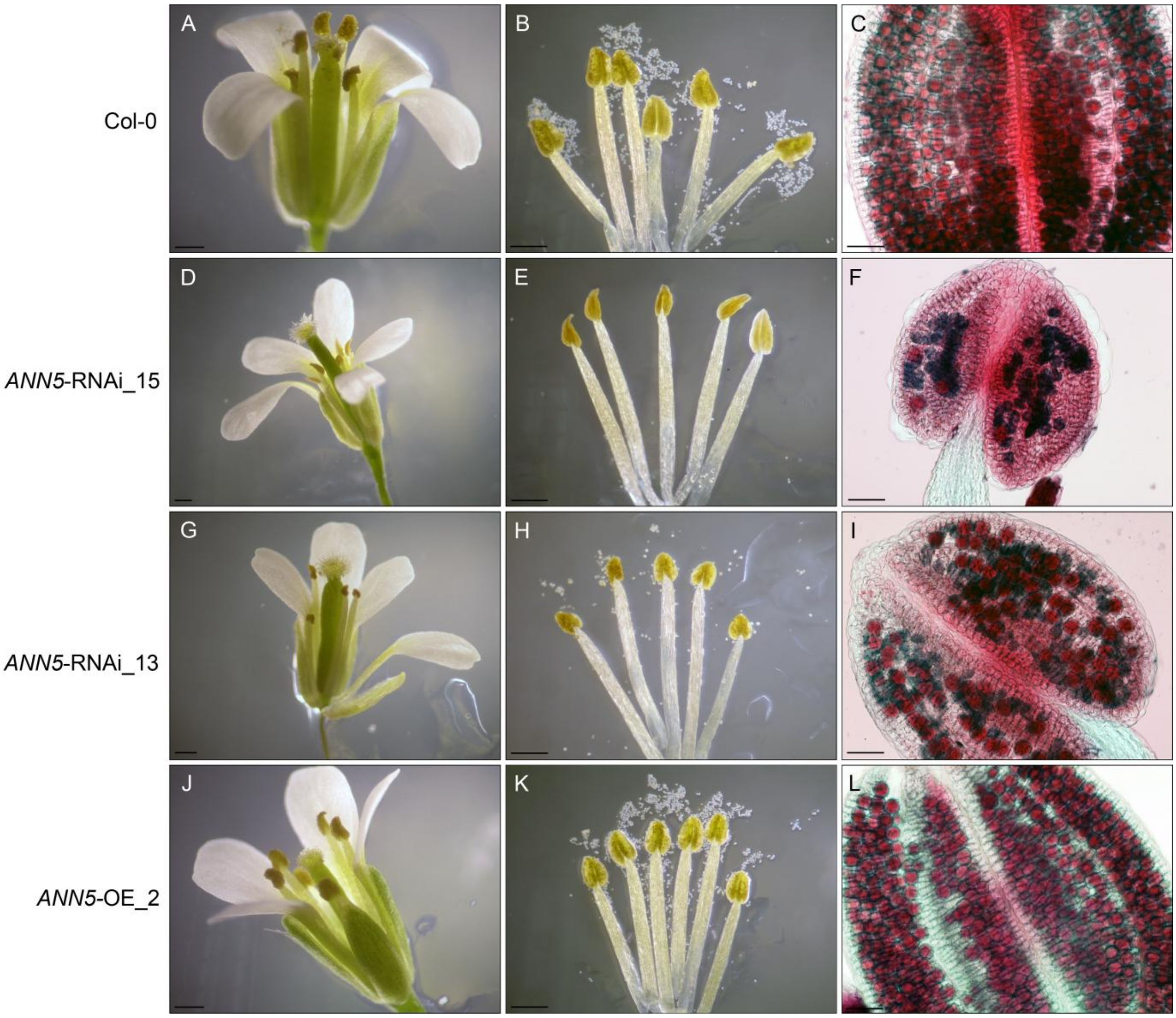

Impact of RNAi-mediated suppression of *ANN5* on pollen viability.

Stereomicroscope images of (A, B, and C) wild-type Arabidopsis (Col-0), (D, E, and F) *ANN5*-RNAi_15, (G, H, and I) *ANN5*-RNAi_13, and (J, K, and L) *ANN5*-OE_2 plants.

Left column (A, D, G, and J), morphology of flowers at anthesis; middle column (B, E, H, and K) dissected stamens; scale bars = 2 mm, and right column (C, F, I, and L) non-dehisced anthers fixed in Carnoy's fixative and stained with simplified Alexander's solution to verify pollen viability [1]. Scale bars = 50 μm.

Figure S4.

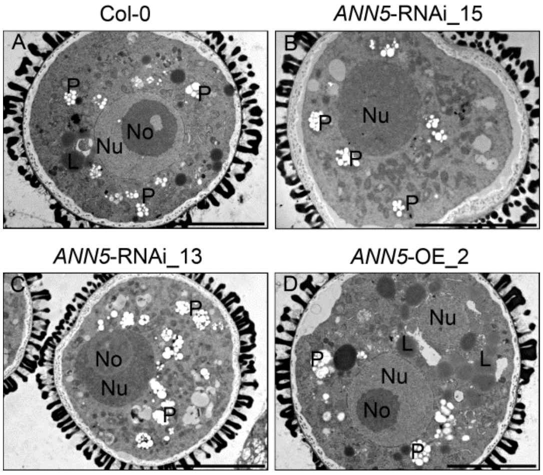

Ultrastructure of bicellular microgametophytes isolated from Arabidopsis lines with altered *ANN5* expression.

Transmission electron micrographs of pollen grains collected from (A) wild-type Col-0, (B) *ANN5*-RNAi_15, (C) *ANN5*-RNAi_13, and (D) *ANN5*-OE_2 plants. Nu: nucleus, No: nucleolus, L: lipid body, P: plastid. Scale bars = 5 µm.

Figure S5.

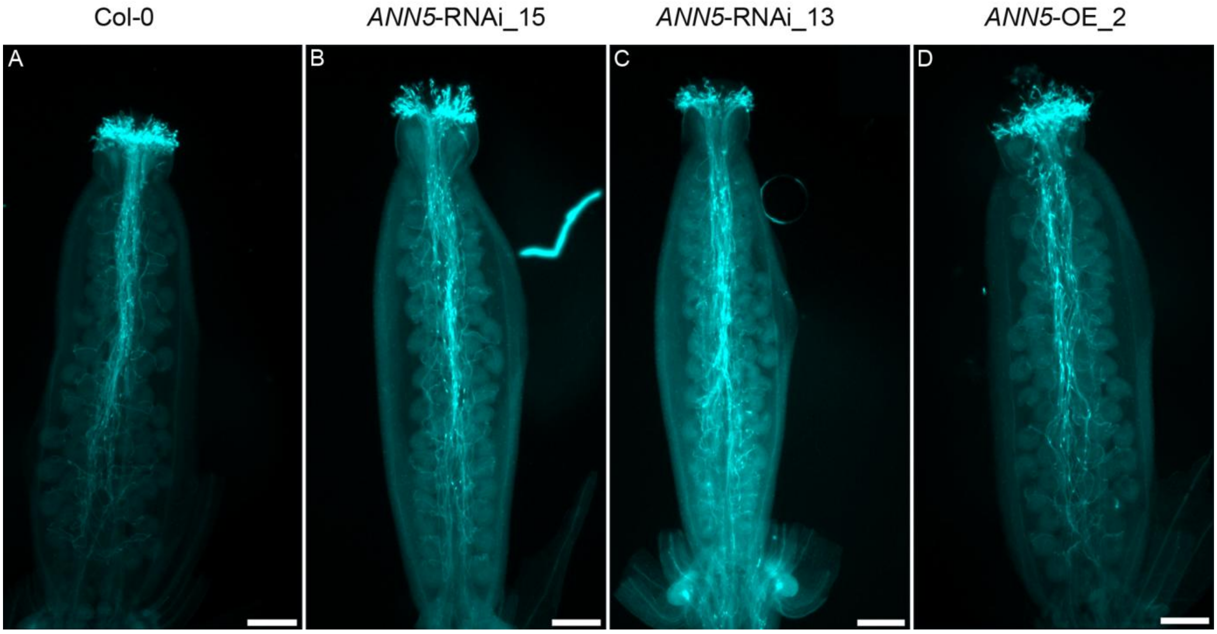

Growth of pollen tubes in pistils 24 h after hand-pollination.

Pollen tubes formed by (A) wild-type Col-0, (B) *ANN5*-RNAi_15, (C) *ANN5*-RNAi_13, and (D) *ANN5*-OE_2 pollen grains were visualized by Aniline Blue staining. Scale bars = 200 μm.

Table S4. List of plastidial proteins co-purified with ANN5-YFP and identified by mass spectrometry.

No	Accession No.	Description	Protein score	Unique peptides	Protein coverage
1	AT4G20360.1	RABE1b, Rab GTPase homolog E1B	1646	15	46.2
2	AT3G26650.1	GAPA, glyceraldehydes 3-phosphate dehydrogenase A subunit	988	15	45.5
3	AT2G28000.1	CPN60A, chloroplast chaperonin 60 alpha	910	13	29.7
4	AT5G38420.1	RBCS2B, Rubisco small subunit 2B	834	8	45.3
5	AT1G42970.1	GAPB, glyceraldehydes 3-phosphate dehydrogenase B subunit	722	14	35.8
6	AT4G24280.1	cpHsc70-1, chloroplast heat shock protein 70-1	650	13	23.1
7	AT5G49910.1	cpHsc70-2, chloroplast heat shock protein 70-2	585	13	23.1
8	AT5G65220.1	Ribosomal L29 family protein	279	4	23.1
9	ATCG00790.1	RPL16, chloroplast ribosomal protein	237	3	31.1
10	AT3G25920.1	RPL15, chloroplast ribosomal protein	191	5	23.8

Proteins are ordered by protein score. Protein score denotes the sum of the scores of the individual peptides calculated by Mascot. Plastidial proteins identified both in the control GFP samples and ANN5-YFP were excluded.

Figure S6.

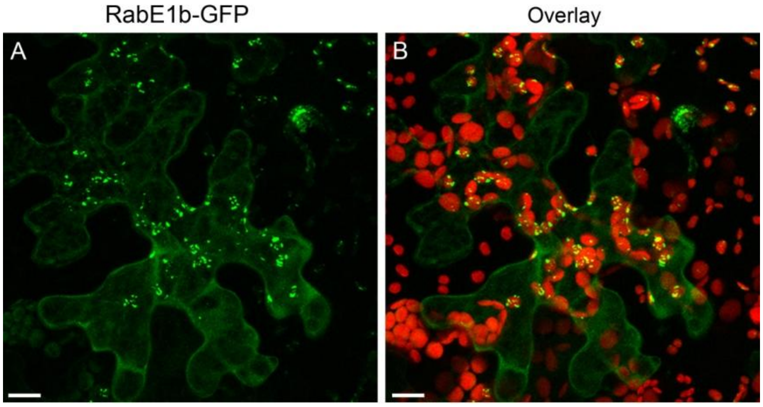

Subcellular localization of RABE1b-GFP in *N. benthamiana* leaf epidermal cells.

(A) Confocal optical section of RABE1b-GFP fluorescence in epidermal cell. (B) Fluorescence of RABE1b-GFP merged with chlorophyll autofluorescence. Scale bars = 10 µm.

Figure S7.

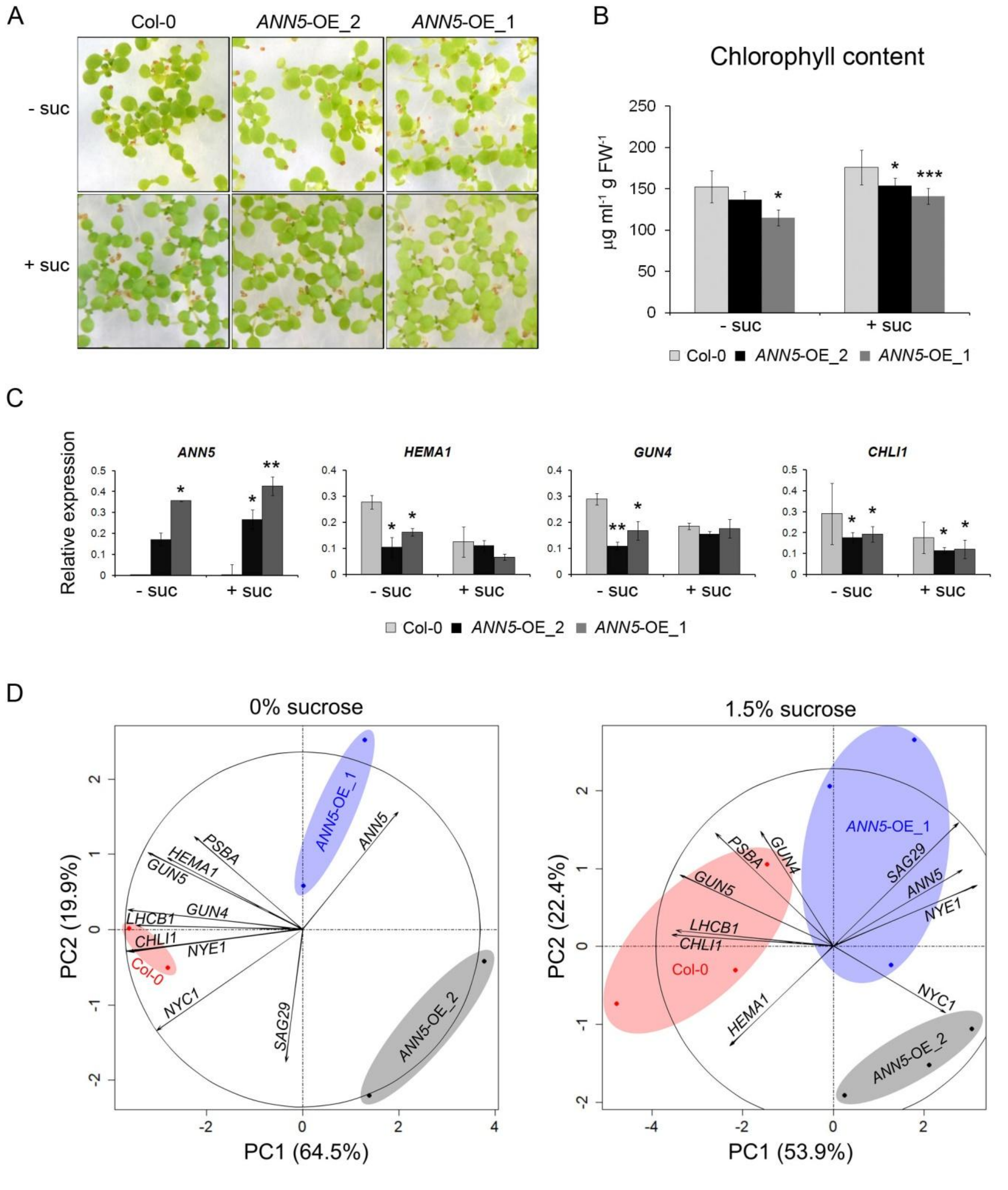

Overexpression of *ANN5* influences chlorophyll content and alters expression of genes related to chlorophyll metabolism in Arabidopsis seedlings.

(A) 10-day old seedlings grown on MS medium in the absence (upper row) and in the presence of sucrose 1.5% (lower row).

(B) Average chlorophyll content in 10-day old seedlings of wild-type and *ANN5* overexpression lines. Three independent experiments were performed with similar outcomes. Asterisks indicate significant difference compared with values for wild-type

seedlings (one-way ANOVA, Dunnett post hoc test, *p < 0.05; **p < 0.01; ***p < 0.001). n=7. Bars represent SD.

(C) Relative expression of *ANN5* in 10-day old seedlings and genes related to chlorophyll biosynthesis (*HEMA1*, *GUN4*, *CHLI1*). Asterisks indicate significant difference compared with values for wild-type seedlings (one-way ANOVA, Dunnett post hoc test, *p < 0.05; **p < 0.01; ***p < 0.001). Bars represent SD.

(D) Principal component analysis performed on gene expression associated with chlorophyll metabolism in 10-day old seedlings grown on MS medium in the absence or presence of 1.5% sucrose. Biplot includes the representations of transcripts and individual samples. All gene expression levels but *ANN5* and *NYE1* were *ln*-transformed prior to the analysis to improve the normality of their distributions. Data was scaled and centered. The analysis was performed using *dudi.pca* function from *R/ade4* package [2].

Chlorophyll biosynthesis: *HEMA1*, glutamyl-tRNA reductase, *GUN4*, encodes a subunit of Mg-chelatase, *GUN5*, encodes a subunit of Mg-chelatase, *CHLI1*, encodes a subunit of Mg-chelatase. Photosynthesis: *PsbA*, encodes a photosystem II chlorophyll binding protein, *LHCB1*, encodes a photosystem II light harvesting chlorophyll binding protein. Chlorophyll degradation: *NYC1*, encodes a chlorophyll b reductase, *NYE1*, senescence-inducible chloroplast stay-green protein, *SAG29*, encodes sucrose efflux transporter, senescence-associated gene.

Supplemental References

1. Peterson R, Slovin JP, Chen C: **A Simplified Method for Differential Staining of Aborted and Non-Aborted Pollen Grains**. *International Journal of plant biology* 2010, **1**(2).
2. Dray S, Dufour A-B: **The ade4 Package: Implementing the Duality Diagram for Ecologists**. In., vol. 22: Journal of Statistical Software; 2007: 1-20.
3. Hilson P, Allemeersch J, Altmann T, Aubourg S, Avon A, Beynon J, Bhalerao RP, Bitton F, Caboche M, Cannoot B *et al*: **Versatile gene-specific sequence tags for Arabidopsis functional genomics: transcript profiling and reverse genetics applications**. *Genome Res* 2004, **14**(10B):2176-2189.
4. Hajdukiewicz P, Svab Z, Maliga P: **The small, versatile pPZP family of Agrobacterium binary vectors for plant transformation**. *Plant Mol Biol* 1994, **25**(6):989-994.
5. Cortleven A, Marg I, Yamburenko MV, Schlicke H, Hill K, Grimm B, Schaller GE, Schmulling T: **Cytokinin Regulates the Etioplast-Chloroplast Transition through the Two-Component Signaling System and Activation of Chloroplast-Related Genes**. *Plant Physiol* 2016, **172**(1):464-478.
6. Huang YS, Li HM: **Arabidopsis CHLI2 can substitute for CHLI1**. *Plant Physiol* 2009, **150**(2):636-645.
7. Gao ZP, Yu QB, Zhao TT, Ma Q, Chen GX, Yang ZN: **A functional component of the transcriptionally active chromosome complex, Arabidopsis pTAC14, interacts with pTAC12/HEMERA and regulates plastid gene expression**. *Plant Physiol* 2011, **157**(4):1733-1745.
8. Gao S, Gao J, Zhu X, Song Y, Li Z, Ren G, Zhou X, Kuai B: **ABF2, ABF3, and ABF4 Promote ABA-Mediated Chlorophyll Degradation and Leaf Senescence by Transcriptional Activation of Chlorophyll Catabolic Genes and Senescence-Associated Genes in Arabidopsis**. *Mol Plant* 2016, **9**(9):1272-1285.
